# In-Plane Si Microneedles: Fabrication, Characterization, Modeling and Applications

**DOI:** 10.3390/mi13050657

**Published:** 2022-04-20

**Authors:** Abdulla Al Mamun, Feng Zhao

**Affiliations:** Micro/Nanoelectronics and Energy Laboratory, School of Engineering and Computer Science, Washington State University, Vancouver, WA 98686, USA; abdullaal.mamun@wsu.edu

**Keywords:** silicon, microneedle, in-plane, drug delivery, sample collection, sensing, CMOS compatible

## Abstract

Microneedles are getting more and more attention in research and commercialization since their advancement in the 1990s due to the advantages over traditional hypodermic needles such as minimum invasiveness, low material and fabrication cost, and precise needle geometry control, etc. The design and fabrication of microneedles depend on various factors such as the type of materials used, fabrication planes and techniques, needle structures, etc. In the past years, in-plane and out-of-plane microneedle technologies made by silicon (Si), polymer, metal, and other materials have been developed for numerous biomedical applications including drug delivery, sample collections, medical diagnostics, and bio-sensing. Among these microneedle technologies, in-plane Si microneedles excel by the inherent properties of Si such as mechanical strength, wear resistance, biocompatibility, and structural advantages of in-plane configuration such as a wide range of length, readiness of integration with other supporting components, and complementary metal-oxide-semiconductor (CMOS) compatible fabrication. This article aims to provide a review of in-plane Si microneedles with a focus on fabrication techniques, theoretical and numerical analysis, experimental characterization of structural and fluidic behaviors, major applications, potential challenges, and future prospects.

## 1. Introduction

Nowadays, the biomedical sector necessitates very small hypodermic needles [1] Conventional hypodermic needles have several disadvantages, such as pain and tissue inflammation induced by insertion, failure to maintain regulated and sustained drug release or sample extraction, and demand for skilled personnel to perform insertion [2], etc. These problems associated with conventional hypodermic needles can be overcome by microneedles [3], which can be fabricated in a variety of sizes and geometric shapes by using photolithography [4]. Microneedles have two main advantages over traditional hypodermic needles. First, the microneedle induces less pain during skin penetration due to its small tip size [5,6]. Second, microneedles can deliver drugs or extract samples from an exact pinpointed location [7]. The concept of the microneedle was first introduced in the 1970s [8], but it was not realized by experimentation until the 1990s [9]. In 1998, the first comprehensive study on transdermal drug delivery was performed by McAllister et al. [10]. Microneedles can be used as a pivotal part in the field of biomedical instrumentation [11] such as transdermal drug delivery [12], body fluid sampling [13], neural stimulation [14], and bio-sensor measurement, etc. [15].

Microneedles are mainly classified into in-plane and out-of-plane microneedles. Out-of-plane microneedles have a limitation of lower penetration depth and difficulty in integrating it with other components such as microfluidics [16,17]. In-plane microneedles also have a problem associated with mechanical strength [18]; however, in-plane microneedles can be fabricated in a wide range of lengths [17] and can easily be integrated with microsensors, microfluidic devices, and microelectrode arrays [11]. Microneedles can also be grouped into solid and hollow microneedles. Solid microneedles are desirable for use as surgical tools for piercing the skin [11] with a coating such as dry powder, drugs, or vaccines [19], while hollow microneedles are desirable for drug delivery [11], sample collection, and mass flow sensor [20], etc. Materials selection is another way to classify microneedles [19]. Metal, silicon (Si), and polymer [21] are the main materials for microneedles so far. Among these materials, Si is a distinctive option because of its strength, wear resistance, non-ductility, non-prone to weariness [22,23], biocompatibility, Young modulus, and high tensile strength [3]. Furthermore, it has been reported that Si demonstrated biocompatibility without in vivo detrimental effects [24]. The fabrication processes with lithography and dry and wet etching have been well established for Si microneedles, which provide precise control over the needle dimensions [23] and are CMOS compatible.

The main design criteria for microneedles include sufficient sharpness to penetrate the skin, length to reach the layer of skin to deliver drugs or extract samples without failure to needle shaft or tips and reliability for multiple penetrations into the skin. Several mechanical safety tests are usually required to evaluate microneedle reliability, such as bending moment, buckling force, insertion force, etc. [2,3,16,19,25,26,27,28]. Finite element analysis by software such as ANSYS, COMSOL, ABAQUS etc., was also applied [1,2,3,11,15,29,30]. Fluid flow is also a design basis for microneedles with parameters such as fluid velocity, flow rate, pressure drop, and pressure to be considered when designing microneedles for drug delivery or sample collection [19]. A successful drug delivery process needs a flow rate between 10 and 100 µL/min [9] with an applied pressure between 10 and 100 kPa [19]. The flow rate is directly proportional to the inner diameter and inversely proportional to the length of the microneedle [31]. The fluid flow analysis or computational fluid dynamic (CFD) analysis has been performed [1,2,9,26,27,29,30,31,32]

This paper provides a comprehensive review of in-plane Si microneedle by exploring various geometric shapes, design criteria, fabrication techniques, mathematical modeling for structural and fluid flow analysis, finite element analysis, current and potential applications, and future prospects.

## 2. Design

**Figure 1:**
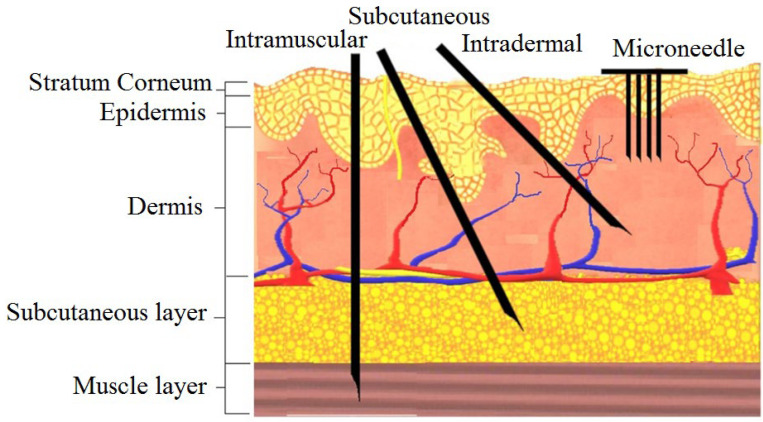
The human skin anatomy and comparison of microneedles with a traditional injectable form of delivery. Reprinted with permission from Reference [38].

The design of in-plane Si microneedles to painlessly penetrate through human skin is closely related to the anatomy of human skin [10]. According to morphology, skin is branched into three strata: epidermis, dermis, and subcutaneous layers [33]. The epidermis consists of stratum corneum (SC) and stratum germinativum (SG) layers [34]. As shown in the anatomy of human skin in Figure 1, blood vessels lie in the dermis layer at the depth of 150~3000 µm from the skin surface [35]. Microneedles should be designed in such a way that they can penetrate the epidermal layer without any infection, pain, or bleeding [36]. The medicinal agent then can permeate into the blood vessels through the dermal layer [37]. The difference between microneedle and traditional transdermal injection is also shown in Figure 1. The diameter of blood corpuscles ranges from 2 µm to 15 µm [35], so for blood flow through microneedle the width and depth (in case of rectangular or square) or diameter (in case of circular) of the inner channel should be greater than 15 µm.

Further design criteria of microneedles are length, width, and thickness which should ensure mechanical stability [11,16]. Free bending force, constrained bending force, buckling force, shear force, compressive force, piercing force, penetration force, etc., play a vital role in determining the mechanical stability of microneedles during human skin penetration [2]. Among these forces, piercing force, buckling force, and bending force have the most impact to determine the overall geometry of the needles [29]. One of the analogies of the microneedle to the natural needle is a female mosquito’s fascicle with a slender, long, and hollow tube that does not break during skin penetration [27]. Various shapes of microneedles have been reported by considering these forces, with some representative designs summarized in Table 1.

## 3. Fabrication

**Figure 2:**
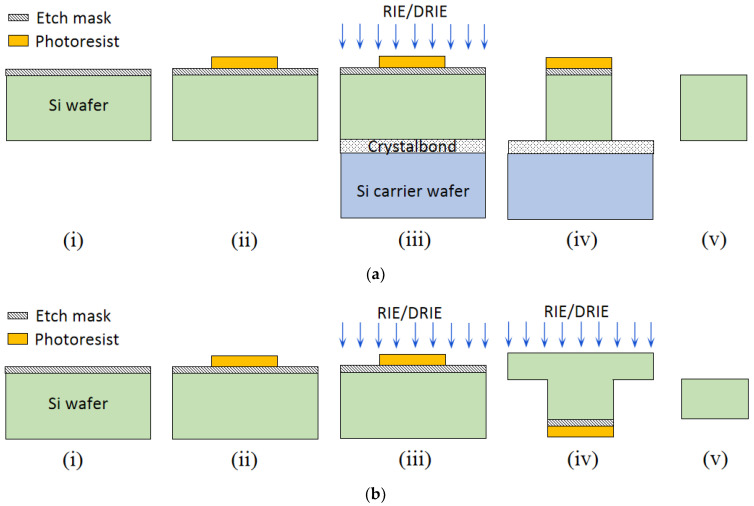
Schematic of two typical fabrication process flows of a 2-D in-plane Si microneedle of (**a**) from only front-side etching and (**b**) both front-side and backside etching. In process flow (**a**), the process steps include: (i) etching mask deposition, (ii) photolithography to pattern in-plane microneedles, (iii) wafer bonding by Crystalbond, followed by RIE or DRIE, (iv) after RIE/DRIE, (v) remove microneedle from Crystalbond and carrier wafer, and remove etching mask and photoresist. In process flow (**b**), (i) etching mask deposition, (ii) photolithography to pattern in-plane microneedles, (iii) front-side RIE or DRIE, (iv) backside RIE or DRIE, (v) microneedle released.

Fabrication techniques of in-plane Si microneedles include deep plasma anisotropic etching, laser machining, electrical discharge machining, 3D printing, magnetization induced self-assembly, thermal drawing, magneto rheological drawing lithography, micro-molding, etc. [15] Among these fabrication techniques, lithography followed by deep plasma etching is well-established and CMOS compatible [1,2,4,11,16,17,20,22,23,25,29,30,32,39,40,41]. This fabrication process typically includes deposition of etching mask material, lithography to pattern microneedle structures, and followed by anisotropic etching by deep reactive ion etching (DRIE) or inductively coupled plasma (ICP) etching. Isotropic wet etching can be applied to sharpen the needle tip [3,42] with a proper etchant such as a mixture of hydrofluoric acid (HF) and nitric acid (H_3_NO_4_). Finally, the suitable metal is coated for improved biocompatibility and the strength of the microneedle. A schematic diagram of in-plane Si microneedle fabricated by two typical process flows is shown in Figure 2.

### 3.1. Etching Mask Materials

An etching mask is required for patterning the in-plane Si microneedles by anisotropic etching. The mask materials need to survive all processes until the end of the fabrication process. Several mask materials have been identified such as Si_3_N_4_ [1,11,17,20] and SiO_2_ [1,2,4,16,22,25,29,30,32]. The deposition of Si_3_N_4_ is mainly by low-pressure chemical vapor deposition (LPCVD) and SiO_2_ by wet or dry thermal oxidation, and the plasma-enhanced chemical vapor deposition process (PECVD) by tetra-ethyl-oxalo-silicate (TEOS).

### 3.2. Pattern Transfer

After etching masking deposition, the next step is pattern transfer by lithography. This process determines the needle geometry including the length, width, and inner channel diameter. There are several techniques for pattern transfer and the most commonly used is lithography, such as standard UV photolithography [1,2,4,16,22,29,39,43], deep UV photolithography [11,17,20,40], a combination of standard photolithography and stepper photolithography [25], ion beam lithography [44,45], electron beam lithography [46], and X-ray lithography [47], etc.

### 3.3. Etching

Etching is the crucial process to get the designed needle shaft and microfluidic channel. Etchant material should be chosen such that the etch rate of Si is greater than the etch mask material. Wet etching, dry etching, and their combination have been used. Tetra-methyl-ammonium-hydroxide (TMAH), Ethylenediamine pyrocatechol (EDP), potassium hydroxide (KOH), HF, and HNO_3_ were typically used for wet etching, while reactive ion etching (RIE) and deep reactive ion etching (DRIE) were used for dry etching.

#### 3.3.1. TMAH Etching

TMAH is an isotropic, wet etching process for the fabrication of in-plane Si microneedles [40]. The etching process is easily controllable and the etch rate does not change over time [48]. This etchant is temperature-dependent and the etch rate of Si in TMAH increases with temperature [49] and concentration to a maximum of 67.62 µm/hr at 4% wt concentration. Furthermore, the etch rate ratio between <111> and <100> is higher than in other orientations [48] therefore a pyramidal tip is achievable.

#### 3.3.2. EDP Etching

EDP is an organic wet chemical etchant and was used for the fabrication of in-plane Si microneedles [25]. The advantage of EDP is that it results in a smooth surface [50]. The etch rate of Si in EDP linearly increases with temperature [50] but decrease with EDP concentration. The etching is limited between 71% and 95% (wt) concentration. Residue starts to form when EDP concentration is below 71% (wt) [50], which limits the etch rate. The better hydrophilic character and very low etch rate of oxide mask [51] make EDP etching widely used in the fabrication of Si MEMS devices and microneedles.

#### 3.3.3. KOH Etching

KOH is relatively less toxic than TMAH and EDP [52]. The etch rate of Si in KOH increases with temperature and is also concentration-dependent. An etch rate of 65 µm/hr and 0.37 µm/hr for Si and SiO_2_, respectively, were achieved in a 45% wt KOH solution [11,17]. When using KOH solution to etch Si with a Si_3_N_4_ mask, it was found that the etch rate of Si does not change significantly with the concentration of KOH from 10% wt to 70% wt [1,20].

#### 3.3.4. HF and HNO_3_ Etching

HF and HNO_3_ mixtures are used for high aspect ratio dependent etching (ARDE). It is a nitric acid dominant wet etchant, for example, HF:HNO_3−_ = 1:19 was used to sharpen the needle tip [3,41]. In static etching, the etch rate of Si in HF and HNO_3_ mixtures is the highest at the needle tip and gradually reduces toward the needle base of the needle due to the less concentration, while in a dynamic etching by stirring the solution, a uniform etching can also be achieved [53]. This etchant solution is also temperature-dependent, as the etching rate increases with temperature [53].

#### 3.3.5. RIE and DRIE Etching

RIE and DRIE are plasma-based dry etching techniques, a combination of physical and chemical processes widely used for the fabrication of in-plane Si microneedles [1,2,3,4,11,17,20,22,29,30,39,41,43,46]. RIE requires special machinery set up such as radio frequency (RF) and vacuum chamber therefore it is a costly etching process in comparison with wet etching, but with a much higher etching rate [54] and anisotropic etching without depending on the crystalline plane. Anisotropy is achieved by etching and sidewall passivation occurring consequently during etching, with sidewall passivation significantly reducing vertical etching rates. DRIE is one type of RIE technique used to achieve deep features with a high aspect ratio.

### 3.4. Coating

Microneedles need to be coated with various materials after fabrication in order to improve biocompatibility and strength. Coating materials include Al and AlN [2,29], metals such as Ti, Pt, and Ni [1,25,30,40], and poly-ethylene glycol di-acrylate (PEGDA) [55].

### 3.5. Tip Sharpening

**Figure 3:**
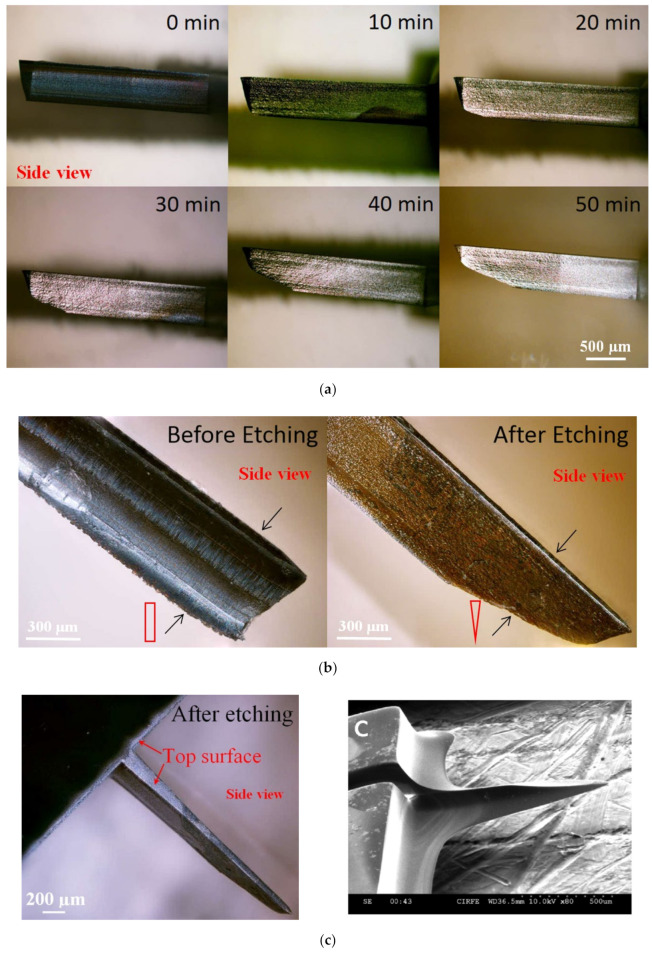
(**a**) Side-view microscopic pictures of the microneedle show the tip sharpening during chemical etching. (**b**) Comparison of the microneedle tip before and after etching by side-view microscopic pictures with cross-section diagrams. (**c**) Microscopic and SEM pictures of microneedles after tip sharpening etching. Reprinted with permission from refs. [3,42].

In order to improve the insertion of the in-plane Si microneedles, the chemical etching process is usually applied to efficiently taper the base and sharpen the needle [3,42]. In these studies, the needle tips were sharpened by a wet chemical etching process in a mixed solution of HF and HNO3 (1:20 by volume). Due to the very small space between the microneedle surface and the Si piece, a concentration gradient of etching species was built up from the top surface to the bottom surface of the needle tip and therefore an etching rate gradient, which leads to the final etching profile that the wedge-shaped tip gradually tapered to the base with a pointed tip formed. The sharpening process of the probe tip before, during, and after wet chemical etching is shown in Figure 3a. Side-view microscopic pictures and cross-section diagrams in Figure 3b compare the microneedle tip before etching (wedge-shaped with rectangular cross-section) and after etching (pointed tip with triangular cross-section) etching. The whole microneedle after etching is shown in the microscopic picture in Figure 3c.

## 4. Analysis

This section deals with theoretical and numerical analyses of the success or failure of the in-plane Si microneedle during skin penetration and computational fluid dynamics for fluid flow through the microneedle, assisted by simulation software such as ANSYS and COMSOL, etc. Some insertion tests are also included in this section.

### 4.1. Theoretical Analysis

#### 4.1.1. Mechanical Strength Analysis

Since silicon is a brittle material, cracks may begin and spread in the microneedles by the induced force during the penetration of the skin and lead to a failure [1]. Six loading conditions are applied for the force analysis of in-plane microneedles.

(a) Buckling Force

Buckling occurs when microneedle pierces human skin [1,2,56]. If the area moment of inertia of the microneedle is not supported by the needle length, the needle will buckle and fail [1,2]. To model the buckling force, the microneedle is considered a fixed joint at the base and a free end at the tip [1,2,16,29,30,31,57]. Buckling stress can be obtained by Euler’s elastic theory [1,2,27] when the needle has a long column, i.e., the slenderness ratio is greater than the critical slenderness ratio [1,27]. The slenderness ratio is given by [1]:

(1) $$\left(\frac{L}{K}\right)=\sqrt{\frac{\pi^{2}EA}{4F_{cr}}}$$

where *E* is the Young’s Modulus, and for Si, *E* = 169 GPa. *A* is the cross-sectional area of the microneedle. *F_cr_* is the critical force for short-column buckling. *K* is the moment of gyration and is defined as [1]:

(2) $$K=\sqrt{\frac{I}{A}}$$

where, *I* is the area/second moment of inertia. The critical slenderness ratio is [1]:

(3) $${\left(\frac{L}{K}\right)}_{cr}=\sqrt{\frac{\pi^{2}E}{2\sigma_{y}}}$$

where *σ_y_* is the yield strength, and for Si, *σ_y_* = 7 Gpa. With the value of *E* and *σ_y_*, the critical slenderness ratio for Si is 11. For a standard Si microneedle, the slenderness ratio is around 114 [1], so Euler’s theory can be applied to determine the buckling force of a microneedle. The maximum buckling force that a microneedle can withstand is defined by [1,2,29,30]:

(4) $$F=\frac{C\pi^{2}EI}{L^{2}}$$

where *C* is a constant with a value of 0.25 when the microneedle is modeled as a fixed-free column. *I* is the area moment of inertia. Table 2 shows the values of *I* for microneedles with different shapes.

**Figure 4:**
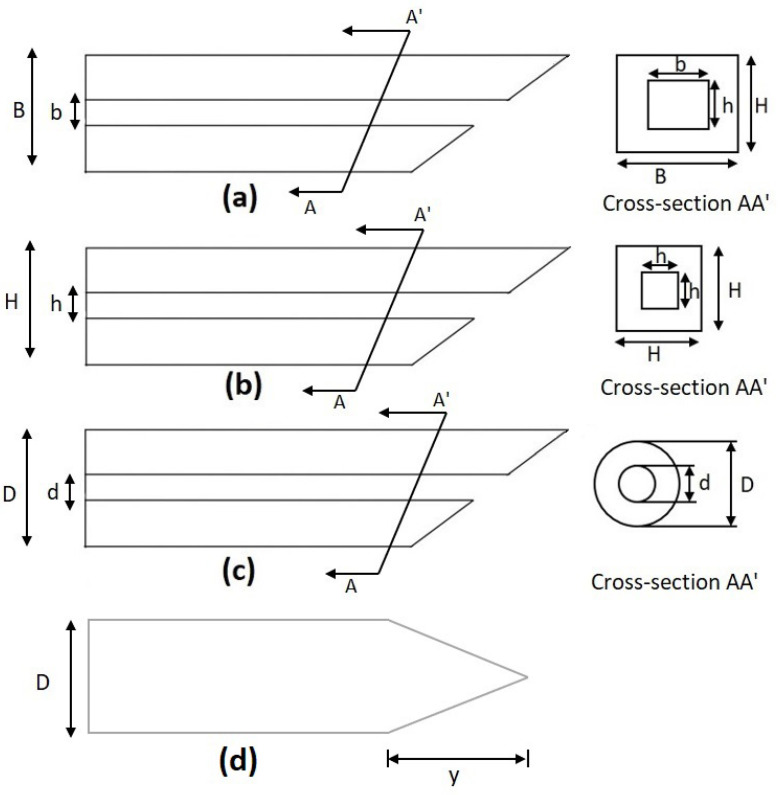
Cross-section of a microneedle design. (**a**) Rectangular, (**b**) square, (**c**) circular, and (**d**) solid conical as listed in Table 2.

**Figure 5:**
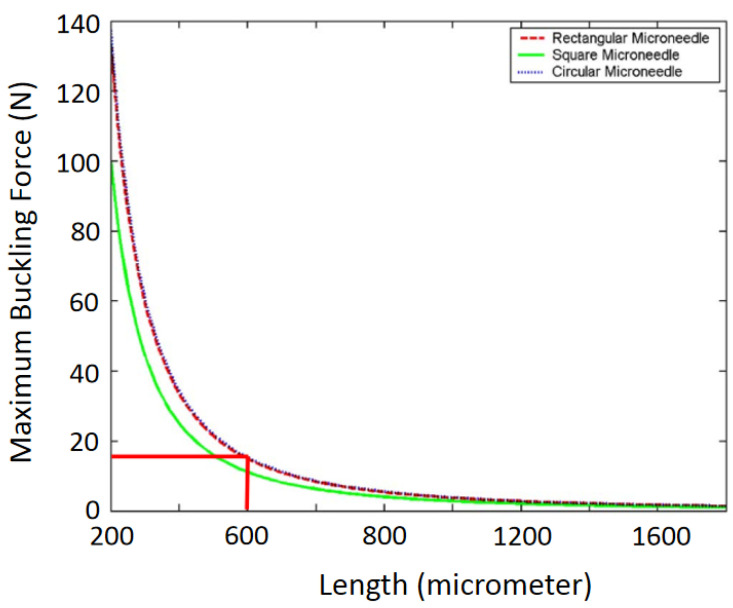
Maximum buckling force as a function of the microneedle length. Reprinted with permission from ref. [2].

The cross-sectional layouts of the typical microneedles in Table 2 are depicted in Figure 4. *D* is the outer diameter, *d* is the internal diameter, and *L* is the length of the microneedle. From Equation (4), we can conclude that the maximum buckling force a microneedle can tolerate is inversely proportional to the square of the length and directly proportional to the cross-sectional area (i.e., the thickness and width of the microneedle or the diameter of the microneedle). This phenomenon has been depicted in Figure 5 [2,58,59]. It is concluded that circular microneedles can tolerate more buckling force than square and rectangular shaped microneedles.

(b) Free Bending Force

When the microneedle is inserted into the skin, it should not experience bending forces if the applied force is perfectly horizontal to the needle shaft. However, the bending force is always generated in real insertion processes [1,60] due to the misalignment of the needle with the skin surface. The induced bending force is analyzed with the microneedle being treated as a cantilever beam [1,2,29,30]. The maximum bending force on a microneedle is defined as [1,2,26,27,29,30,56]:

(5) $$F_{Bending}=\frac{\sigma_{y}I}{cL}$$

where *c* is the normal distance between the neutral axis to the outer edge of the microneedle [2,19,29] *c* = *H*/2 where *H* denotes the external dimension of the needle. For rectangular and circular cross-sections, *H* is the thickness and diameter, respectively [2].

**Figure 6:**
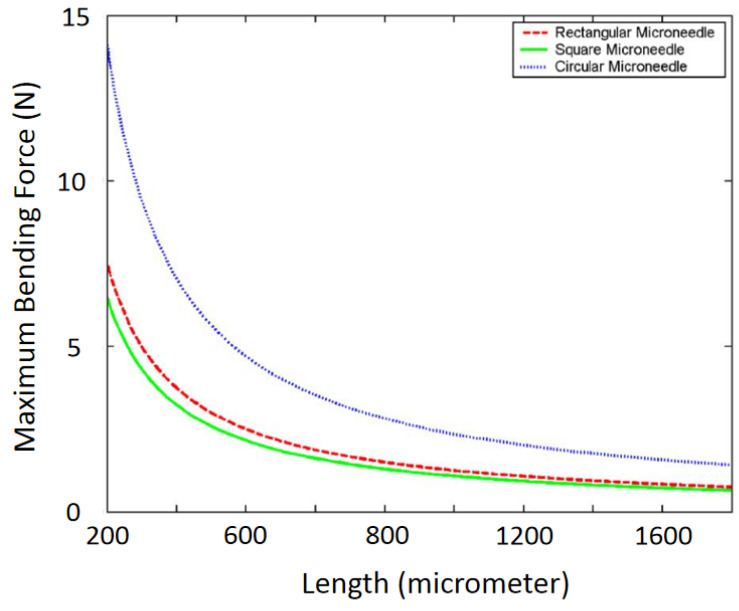
Variation of maximum bending force with the length of a microneedle. Reprinted with permission from ref. [2].

If a microneedle is coated with Ni, Pt, or Au, etc., the maximum bending force is [1]:

(6) $$F_{Bending}=\frac{\sigma_{y}\left(E_{Si}I_{Si}+E_{Mt}I_{Mt}\right)}{C_{Si}LE_{Si}}$$

where *E_Si_* and *E_Mt_* are Young’s Modulus of silicon microneedle and metal coating, respectively. Equation (5) shows that the maximum bending force is proportional to needle thickness and width but inversely proportional to needle length. An example of the variation of the maximum bending force with the microneedle length is shown in Figure 6 [2,29].

(c) Constrained Bending Force

When a microneedle is inserted into the skin, it can no longer move freely; instead, it undergoes constrained movement that leads to a constrained bending force [2,30]. The maximum constrained bending force is determined by [2,30] Equation (7), which shows that the maximum constrained bending stress is two times that of the free bending stress as in Equation (5).

(7) $$F_{MaxConstrainedBending}=\frac{2\sigma_{y}I}{cL}$$

(d) Compressive Force

When the microneedle is penetrated into the skin by applying axial or horizontal force, it experiences a compressive force which causes buckling of the needle [2,19,30]. The maximum compressive force is defined [2,19,26,30] as:

(8) $$F_{MaxCompressive}=\sigma_{y}A$$

where *A* denotes the cross-sectional area of the microneedle.

(e) Shear Force

Microneedles experience perpendicular movement between the base and the tip when it completely penetrates the skin, which leads to shear stress [1,2,30]. The maximum shear stress is given by [1,2,30]:

(9) $$F_{MaxShear}=\frac{\sigma_{y}A}{2}$$

Equations (8) and (9) indicate that the maximum compressive force is two times the maximum shear force.

(f) Penetration Force

Human skin exerts a resistive force on the microneedle when the microneedle penetrates through the skin [2,30]. This resistive force is calculated by [2,19,26,30]:

*F_resistance_* = *P_pierce_A* (10)

where *P_Pierce_* denotes the pressure by the skin on the microneedle during penetration, and it is reported to be 3.18 MPa [2,61]. When the needle is inserted inside the skin, the resistive force reduces remarkably [62,63]. Variation of skin resistance with the thickness of the microneedle is depicted in a reference [29].

**Figure 7:**
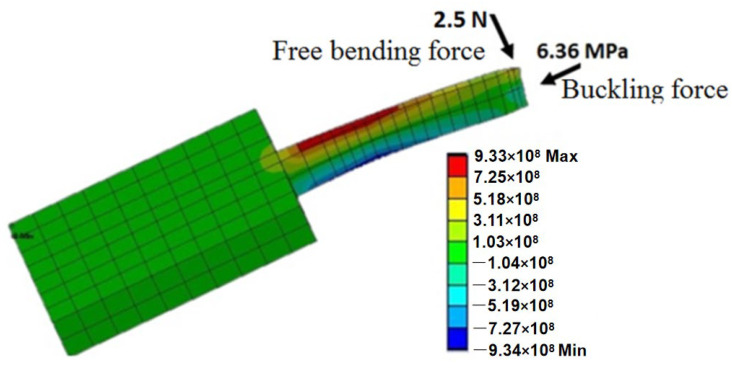
Free bending force and buckling force acting on the microneedle. Reprinted with permission from Reference [42].

Among all six types of forces, it has been found that the maximum free bending force and maximum buckling force are the minima [2]. If a microneedle can withstand these two forces, usually it will be strong enough for skin piercing. The directions in which the bending and buckling forces act upon the microneedle are depicted in Figure 7.

#### 4.1.2. Microfluidic Analysis

One of the main applications of microneedles is to draw blood or inject liquid drugs into the skin. The fluid flow through the microneedle depends on various features such as the needle shape, fluid density, fluid viscosity, etc. [19]. For efficient drug supply or sample collection, it is crucial to analyze the microfluidic flow characteristics so that the needle can be sufficiently small to minimize pain but still wide enough for the required fluid flow.

The fluid flow is characterized by the Reynold number (*R_e_*). When *R_e_* < 2100, the flow is laminar otherwise it is turbulent. The Reynold number is defined as [1]:

(11) $$R_{e}=\frac{UD_{h}}{\nu }$$

In this equation, *U* is the mean flow velocity throughout the needle. *υ* is the kinematic viscosity and *υ* = *µ*/*ρ*, where µ is dynamic viscosity and *ρ* is the density of the fluid. *D_h_* is the hydraulic diameter and *D_h_* = 4*A_cs_*/*perimeter*, where *A_cs_* is the cross-sectional area of the microfluidic channel. By replacing *υ* with *µ*/*ρ*, Equation (11) changes to

(12) $$R_{e}=\frac{U\rho D_{h}}{\mu }$$

Fluid velocity is modeled (Zahn et al. 2000) in *x*-direction through a microneedle with a rectangular cross-section (*y*, *z*) as:

(13) $$\begin{array}{c} V_{x\left(y,z\right)}=\frac{16a^{2}}{\mu \pi^{3}}\left(-\frac{dP}{dx}\right)\overset{\infty }{\underset{i=1,3,5\ldots \ldots }{\sum }}{\left(-1\right)}^{\frac{\left(i-1\right)}{2}}\left[1-\frac{cosh\left(\frac{i\pi z}{2a}\right)}{cosh\left(\frac{i\pi b}{2a}\right)}\right]\frac{cos\left(\frac{i\pi y}{2a}\right)}{i^{3}}\\ -a\leq y\leq a\\ -b\leq z\leq b \end{array}$$

where 2*a* and 2*b* are the width and thickness of the microneedle, respectively. By integrating Equation (13) with respect to the *y* and the *z* axis, the average fluid velocity *U* is:

(14) $$Q=\frac{4ba^{3}}{3\mu }\left(-\frac{dP}{dx}\right)\left[1-\frac{192a}{\pi^{5}b}\sum_{i=1,3,5\ldots \ldots }^{\infty }\frac{\tanh(i\pi b/2a)}{i^{5}}\right]$$

(15) $$U=\frac{Q}{4ab}$$

In Equations (13) and (14), −*dP*/*dx* denotes the pressure drop in the microneedle along the *x*-axis. The pressure drop can be categorized into three pressure losses along the microneedle: loss where fluid enters the channel, loss on the channel wall due to viscous drag, and losses due to the specific geometry of the microneedle. Pressure drop through a rectangular microneedle is:

(16) $$\frac{dP}{dx}=-\frac{4\tau_{S}}{D_{h}}$$

where *τ_S_* is identified as:

(17) $$\tau_{S}=\frac{0.332\mu U}{x}\sqrt{R_{e_{x}}}$$

where *x* is the distance along the plate and *R_ex_* is the Reynolds number based on this distance.

By integrating Equation (16), the pressure drop at the entrance is given as (Zahn et al. 2000):

(18) $$\Delta P_{entrance}=\int_{0}^{l}\frac{4}{D_{h}}\tau_{s}dx=\frac{4\times 0.332}{D_{h}}\sqrt{\rho \mu U^{3}}\int_{0}^{l}x^{-\frac{1}{2}}dx=\frac{8\times 0.332}{D_{h}}\sqrt{\rho \mu U^{3}l}$$

*l* is the entrance length of the fluid channel on the microneedle. This entrance length is the distance fluid flows until the pressure gradient is matched with the fully developed flow, and it is given as:

(19) $$\frac{l}{D_{h}}=0.59+0.055R_{e}$$

Pressure drop due to viscous drug at the channel wall is defined by modified Bernoulli’s equation:

(20) $$\Delta P=\Delta P_{ent}+\frac{1}{2}\rho \left(U_{2}^{2}-U_{1}^{2}\right)+f\frac{L}{D_{h}}\frac{1}{2}\rho U^{2}+K_{geom}\frac{1}{2}\rho U^{2}$$

where *L* is the needle length minus the entrance length, *f* is the fraction factor which is given by:

(21) $$f=\frac{4\left|\Delta P_{dev}\right|}{\rho U^{2}}\frac{D_{h}}{L}$$

in which ∆*P_dev_* is the fully developed pressure factor. *K_geom_* is a geometric loss factor. When the needle bends, *K_geom_* = 1.3, and for a sudden contraction:

(22) $$K_{geom}=\left(\frac{2}{m}-\frac{A_{2}}{A_{1}}-1\right)$$

*m* is the root of the quadratic given as:

(23) $$\frac{1-m\left(A_{2}-A_{1}\right)}{1-{\left(A_{2}-A_{1}\right)}^{2}}={\left(\frac{m}{1.2}\right)}^{2}$$

where *A*_1_ and *A*_2_ are the areas of the cross-section before and after contraction, respectively.

### 4.2. Computational Analysis

Computational analysis by the finite element method using simulation software such as ANSYS and COMSOL is critical to assisting the design of microneedles by modeling and analyzing the mechanical strength and fluid dynamics.

#### 4.2.1. Structural Analysis

**Figure 8:**
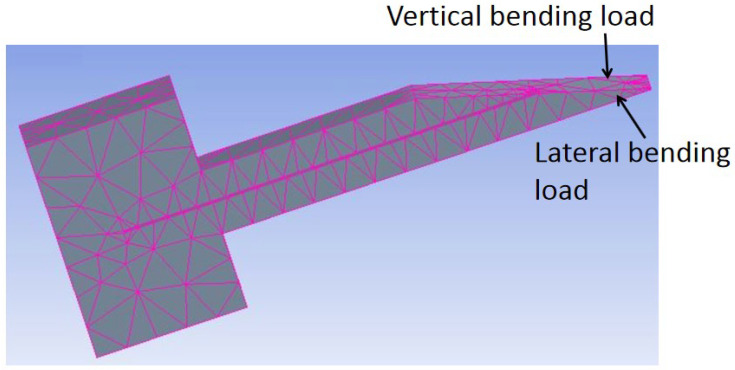
In-plane Si microneedle under vertical and lateral bending load in ANSYS.

Modeling by ANSYS [1,2,11,19] and COMSOL [30] has been performed to analyze the effect of tip loads on microneedle structure and mechanical strength. The bending load and axial load are depicted in Figure 8. Von Mises stresses which take into account all types of possible stress by determining the complete strain at any given point have been calculated by ANSYS [1,11]. It was found that, for axial load, stress decreases sequentially from tip to far end because stress is distributed over a wider cross-section, while for bending load, the maximum stress occurs at the tail end of the microneedle as the bending moment increases linearly from the tip to the tail end of the microneedle.

**Figure 9:**
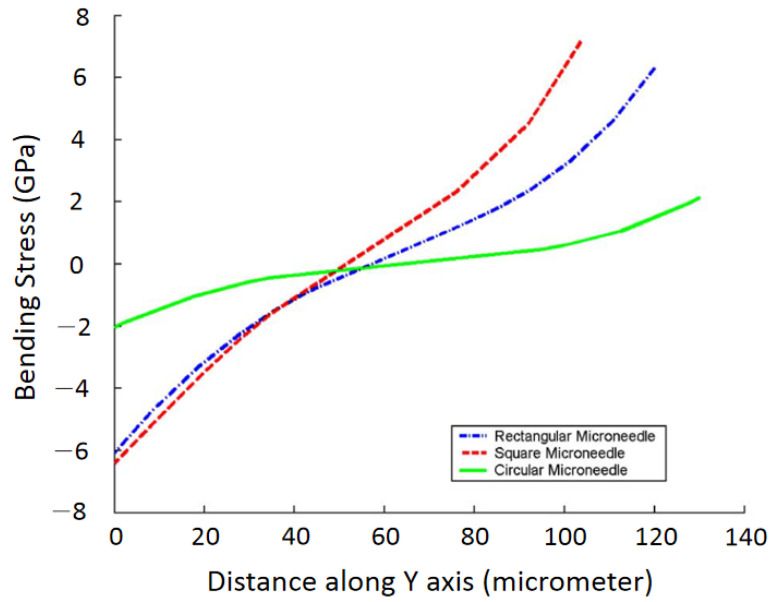
Finite element analysis of bending stress for different shapes of microneedles. Reprinted with permission from Reference [2].

Finite element SOLID 186 in ANSYS was also applied to model microneedles [2] with various geometric shapes of rectangular, circular, and square with the same dimension. It was concluded that under the same bending load, the bending stress in microneedles with circular, square, and rectangular cross-sections increases, respectively, as shown in Figure 9.

#### 4.2.2. Computational Fluid Dynamics

To verify the theoretical analysis of fluid flow characteristics, a computational fluid dynamics analysis by ANSYS and COMSOL has also been performed and reported [1,11,19,26,30]. Aggarwal et al. [64] simulated microneedles for two different lengths and the same cross-sectional area, and for each needle, they found the same characteristics. That is, at any cross-section of the needle, the velocity profile is parabolic where velocity is minimum near the wall and maximum at the center as like laminar flow, the flow remains constant all over the channel except at the inlet and outlet, [1] also got similar result during their simulation. By analyzing their simulations, it can be concluded that the velocity of fluid decreases with the increasing length of the microneedle.

The important findings of numerical simulations in [19] verified theoretical analysis can be summarized as:

- The flow rate increases with inlet pressure;
- The flow rate is slightly less in numerical analysis than in theoretical analysis. It is because frictional losses were not considered during numerical analysis;
- Pressure drop increases with flow rate and inlet pressure.

### 4.3. Experimental Analysis

Experimental analysis by insertion tests have been performed to characterize the potential in vivo behaviors and sustainability of microneedles [1,3,11,15,16,17,23,32,34,39,65,66,67,68,69,70,71]. In this section, we will summarize the experimental analysis of mechanical strength and fluid flow in in-plane Si microneedles.

#### 4.3.1. Mechanical Strength

**Figure 10:**
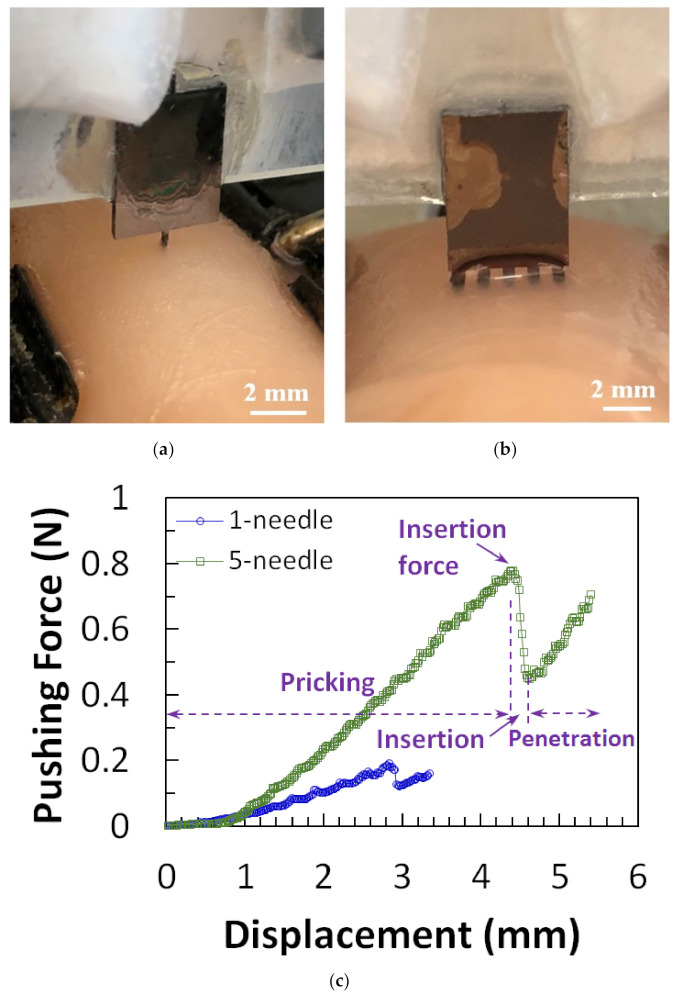

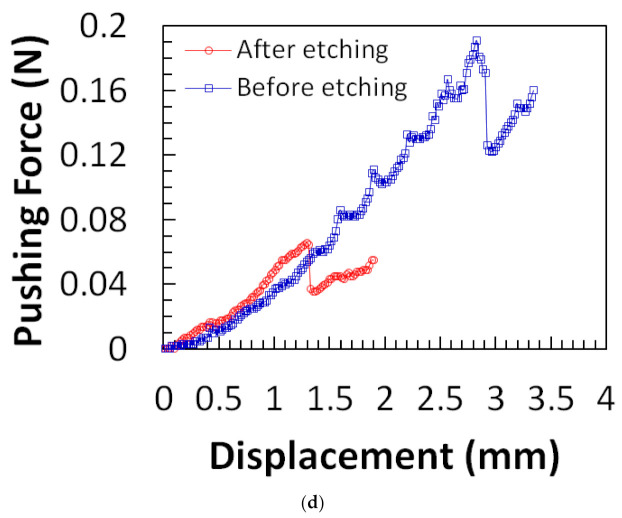
Micrograph of (**a**) one-needle and (**b**) five-needle device in insertion test. (**c**) Force-displacement curves from one-needle and five-needle devices of design. (**d**) Force-displacement measurement results for microneedle before and after tip sharpening. Reprinted with permission from Reference [42].

Distinct tip designs of microneedles have been tested [11,17,42] by piercing the needle into materials such as chicken breast flesh or agarose gel which mimics human skin [72] to determine the penetration strength for insertion. In our recent study [42], it was observed that the insertion force of one-needle devices increased with the tapered angle of needle tips, while five-needle devices required a large insertion force due to the large pricking area. Furthermore, the insertion force, free bending force, and the maximum buckling force were all reduced, and the maximum bending stress was improved after microneedle tip sharpening. The test setup for microneedles on chicken breast flesh and the results are shown in Figure 10.

**Figure 11:**
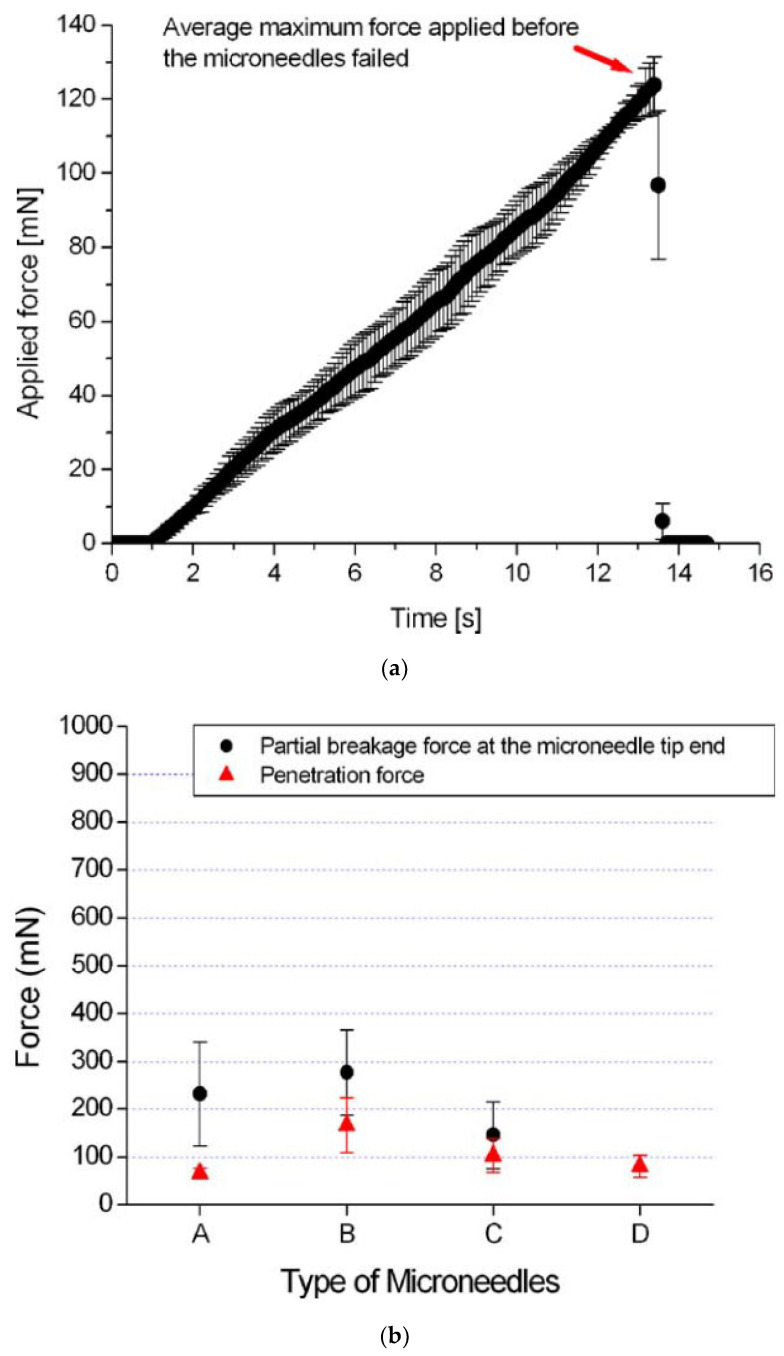
(**a**) Applied force on microneedle shaft. The sudden drop in the force at 124 mN means that the microneedle shaft is broken. The error bars represent the standard deviation of the data around the mean; (**b**) The average force causing significant damage at the tip end and the average penetration force for different types of microneedles. The circle symbol (●) represents the average force that causes severe damage at the tip end of the microneedle shaft of types A, B, and C. The triangle symbol (▲) represents the average penetration force to the chicken breast flesh for different types of microneedles. The error bars represent the standard deviation of the data around the mean. The error bars represent the standard deviation of the data around the mean. Reprinted with permission from Reference [16].

**Figure 12:**
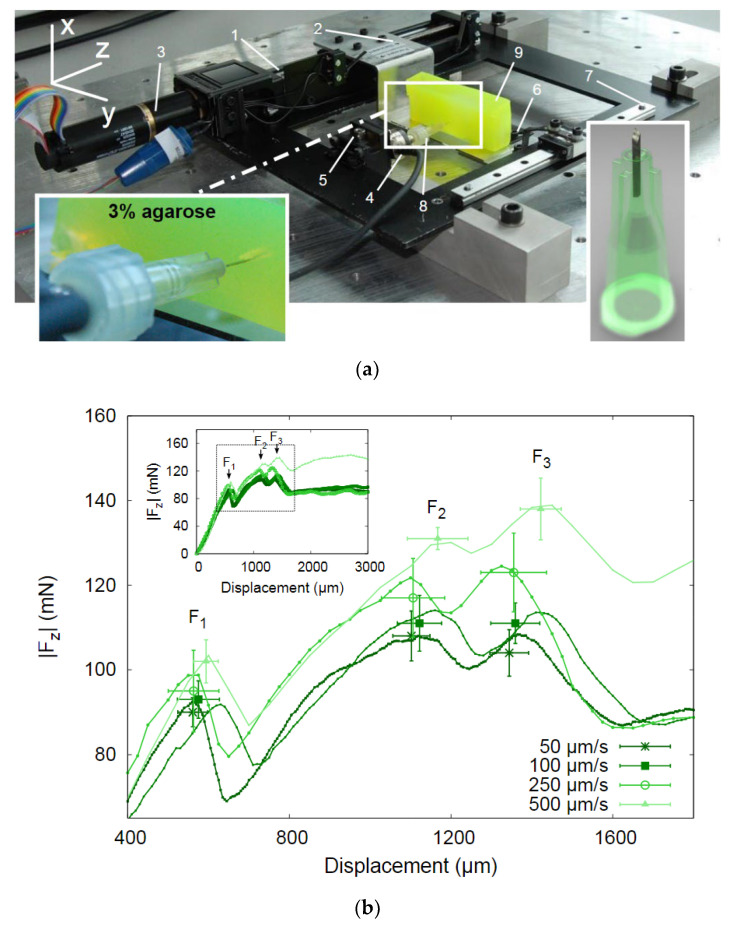

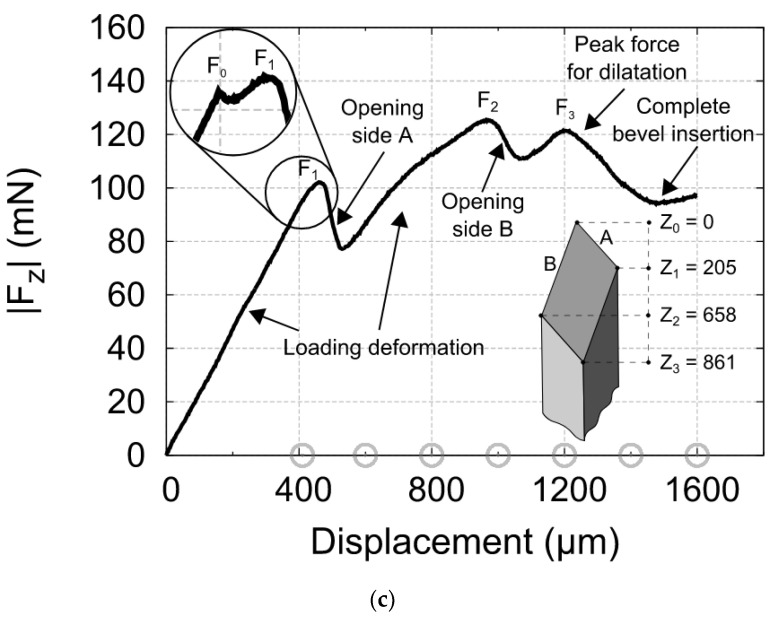
(**a**) Photograph of the insertion force—displacement measurement setup. The important parts are marked: (1) the linear actuator, (2) carriage for holding samples, (3) EC-motor driving the linear actuator, (4) force sensor, (5) adjustable stage to alter the position of the needle in the *x*- direction, (6) braces to hold the sample in place, (7) linear guide to prevent bending and motion of the carriage in *x*- direction, (8) needle, and (9) sample; (**b**) Insertion force- displacement curves of microneedles in 3% agarose gel with polyurethane foil at different speeds. Each curve represents a typical single experiment at one particular speed; the data points are the mean values of the F_1,_ F_2,_ and F_3_ peaks for nine insertion curves including error bars showing the standard deviations; (**c**) Force- displacement curve of a micro-needle insertion experiment. The force characteristics are indicated in the graph. The encircled inset shows that an additional small peak appears if a blunt needle tip is used. The gray circles on the *x*-axis indicate the amount of displacement of other insertion experiments in the series [23].

Besides chicken breast flesh, insertion tests on agarose gel, rabbit, or mouse skins were also performed following the same procedure to characterize the penetrability of microneedles [16,23,65,73]. It was found by a test on 1% agarose gel [16] that the tip with a 30° tip angle is the most vigorous shape. The results are shown in Figure 11. Other insertion tests were performed on a 3% agarose gel covered by an 80 µm polyurethane foil to mimic the dermis and the stratum corneum of human skin. The results show that the insertion force increases with insertion speed, and, after complete insertion, the required force decreases dramatically. The experimental setup and results are shown in Figure 12.

#### 4.3.2. Experiment on Fluid Flow

**Figure 13:**
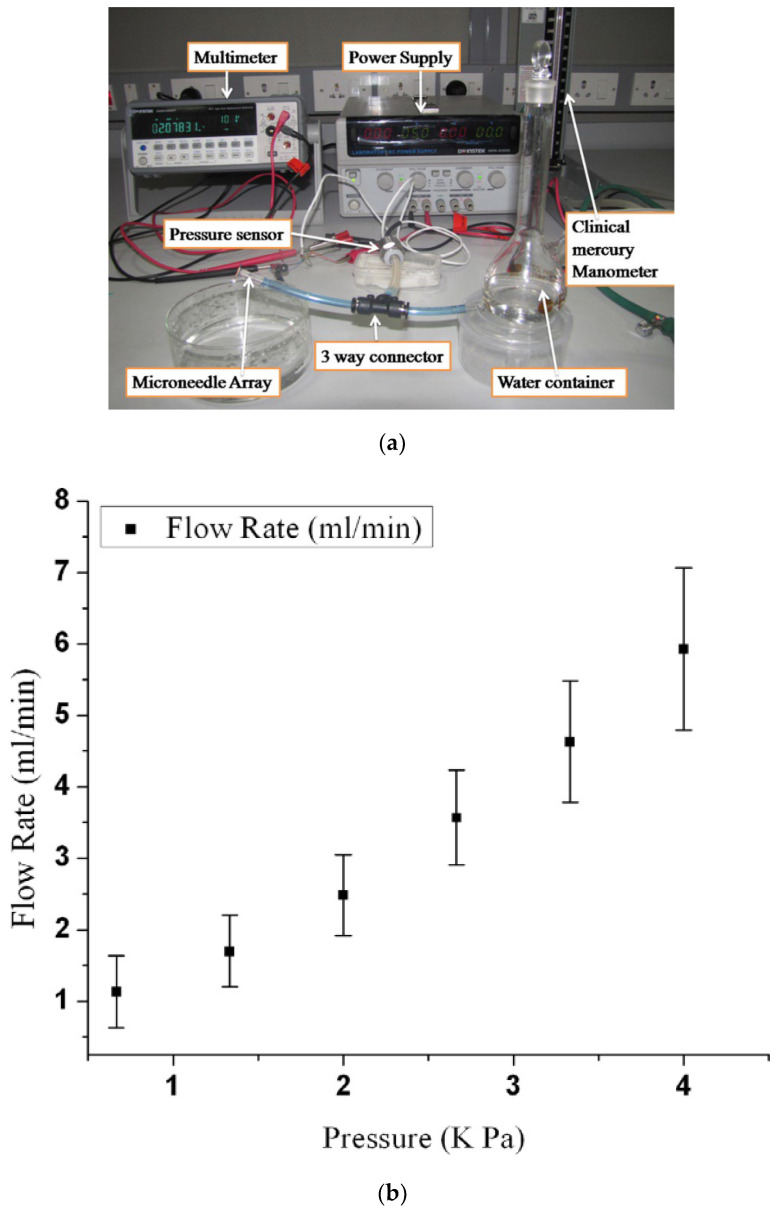
(**a**) Experimental setup used for the characterization of fluid flow in an array of microneedles; (**b**) fluid flow rate versus inlet pressure. Reprinted with permission from ref. [69].

Characteristics of fluid flow through the microneedle to the skin have been investigated vastly [1,3,16,32,39,69,74,75]. The experimental setup for the study requires an air pressure source, pressure regulator, syringe, pump, pressure vessel, pressure gauge, etc. A typical setup is shown in Figure 13 [69]. The findings from the study of [1] are summarized in Table 3.

**Figure 14:**
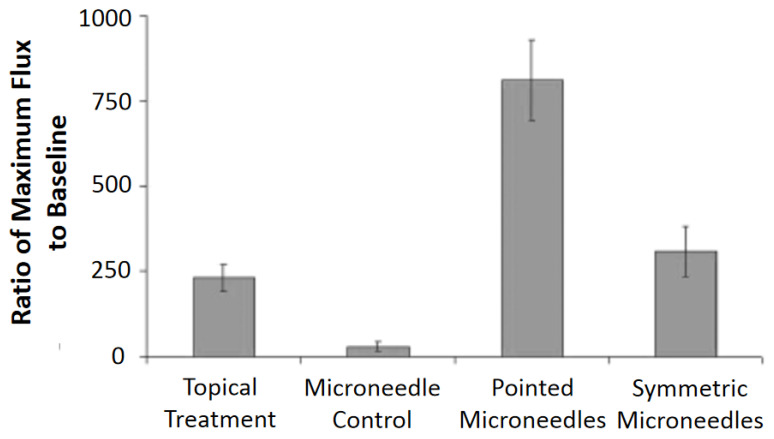
Percent increase over baseline at maximum blood flux: Following 30 s treatments with methyl nicotinate, maximum blood fluxes were compared with baseline blood fluxes, and pointed microneedles significantly increased the maximum blood flux over topical and symmetric microneedle treatments (*p* < 0.05). The microneedle control consisted of an empty microneedle syringe that was pressed into the skin. Reprinted with permission from ref. [75].

Figure 13a shows a setup [69] used to study the fluid flow using H_2_O. It was found that the flow resistance decreases with the increased number of microneedles in an array, and the flow rate increases with inlet pressure as shown in Figure 13b. This observation is consistent with theoretical and numerical analysis. Fluid characteristics of a microneedle with a two-channel probe-shaft have also been studied [32]. A flow of 1.5 µL/min with 1 kPa inlet pressure can be attained by an inner channel diameter of 25 µm. Another study suggested [75] that fluid flows more efficiently in microneedles with a sharply pointed tip than in those with symmetrical tips. Figure 14 shows the graph of the experimental results.

## 5. Applications

In this section, some typical applications of in-plane Si microneedles are summarized.

### 5.1. Drug Delivery

Transdermal drug delivery [1,4,39,69,76,77] is one of the major applications since microneedles provide the best means for regulated and continuous delivery over a controlled period [78,79], at the pin-pointed location [80] or on the body part where the fway toral dosage is not accessible [81], and with minimum side effects [81,82]. Microneedles are easy to use, secure, and can be sharpened for minimal-pain insertion [83]. In 1970, the microneedle was proposed as a drug delivery medium [37], and, in 1976, a patent was filed for the first time [84] by Gerstel et al. [8] on the microneedle-based drug delivery method. Then, after a long break, a microneedle-based drug delivery system was proposed in 1998 [10], followed by numerous researches for efficient drug delivery [26,37], and as a result, it has reached a promising stage of drug delivery to humans [37]. Some drugs reached clinical trials for the delivery by microneedles, such as Naltrexone [85] and ZP-PTH [86]. In 2009, two microneedle-based drugs became available in the market, *Micron-Jet* which consists of four hollow silicon microneedles, and *Soluvia*, a microinjection system with a 1.5 mm long Si microneedle for an influenza vaccine [37,87]. Other microneedle-based drugs are on the way to becoming available in the market. The studies from [59,88,89,90] show that microneedles can successfully deliver proteins, oligonucleotides, insulin, DNA, and RNA into human skin. Microneedles have not only shown efficacy in drug delivery but also have proved their effectiveness with lower doses than regular doses. For example, 50% doses of the hemagglutinin inhibition antibody delivered by microneedles have shown the same result as regular doses [77].

Besides drug delivery, microneedles have also shown success in the treatment of skin cancer [91], dermatitis [92], and glaucoma [7], etc. In addition, capillary blood flow or biological fluid flow analysis through microneedles [13,41,64,73,93,94,95,96] have demonstrated the viability of microneedles for blood analysis, blood sampling, or interstitial fluid sampling for diagnostics.

### 5.2. Bio-Signal Monitoring

Bio-signal monitoring is crucial for human health examination and early disease diagnostics such as Parkinson’s disease [14]. Among all bio-signals, three are the most important to realize the physiological and pathological state of human health: electrocardiography (ECG), electromyography (EMG), and electroencephalography (EEG), which correspond to the signals of the heart, brain, and muscle, respectively [15,97,98,99]. Medical electrodes such as the conventional wet electrode (Ag/AgCl) or microneedle array electrode (MAE) can effectively measure these bio-signals [100]. The wet electrode has a limitation of skin irritation or allergic reactions [101] and needs skin preparation [102], which is not suitable for long-term use [103]. MAE or dry electrode with a coating of titanium (Ti), gold (Au), silver (Ag), or silver-chloride (AgCl) is a successful alternative [15].

**Figure 15:**
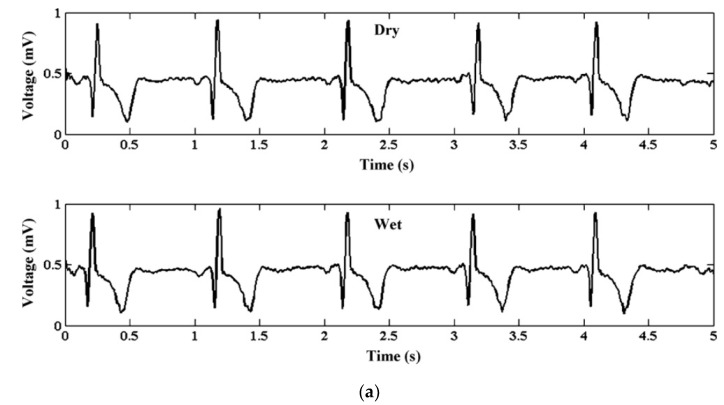

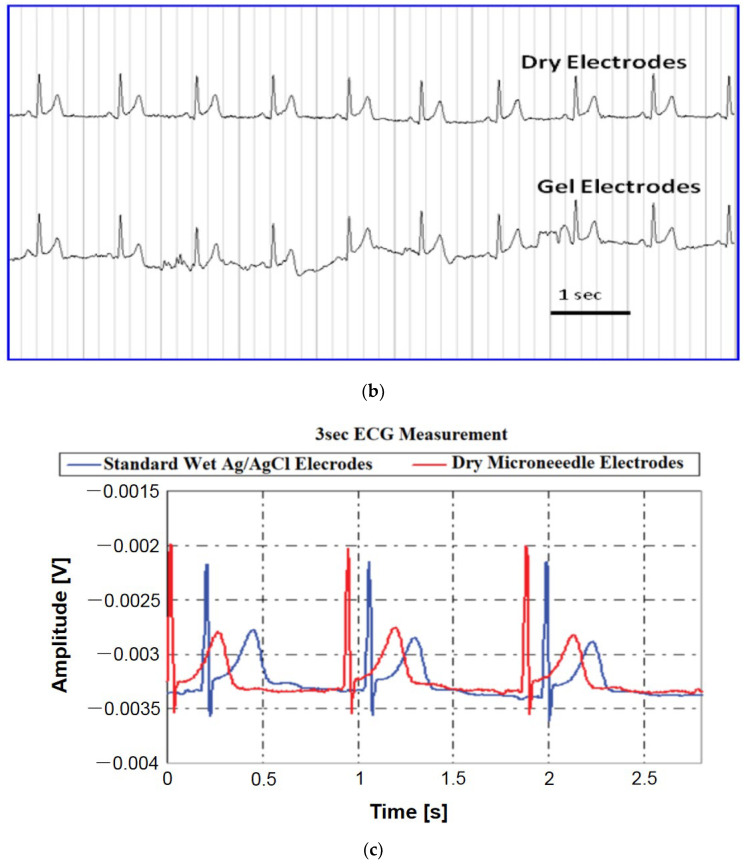

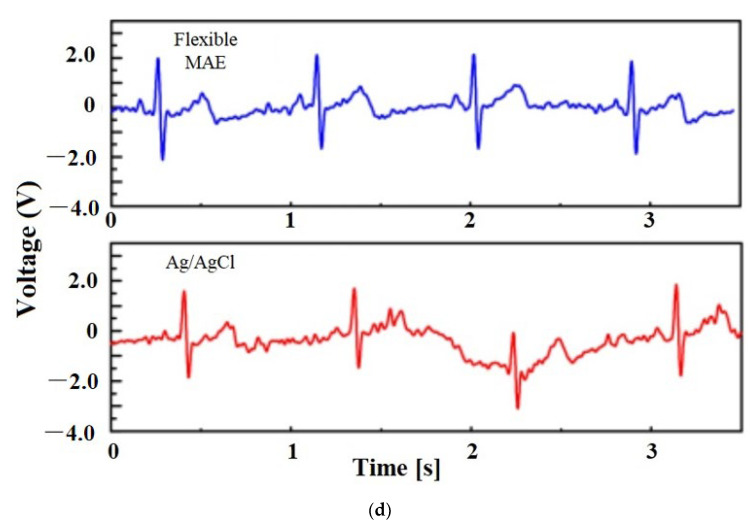
(**a**) ECG from dry and wet electrodes. Reprinted with permission from ref. [108] (**b**) ECG data of the dry and wet gel-based electrodes after 2 h with the subject in moving condition. Reprinted with permission from ref. [103]; (**c**) ECG measurements from dry electrodes are in close agreement with those obtained using standard wet electrodes. Reprinted with permission from ref. [112]; (**d**) ECG signals recorded by the Ag/AgCl electrode and flexible MAE in the dynamic state [111].

**Figure 16:**
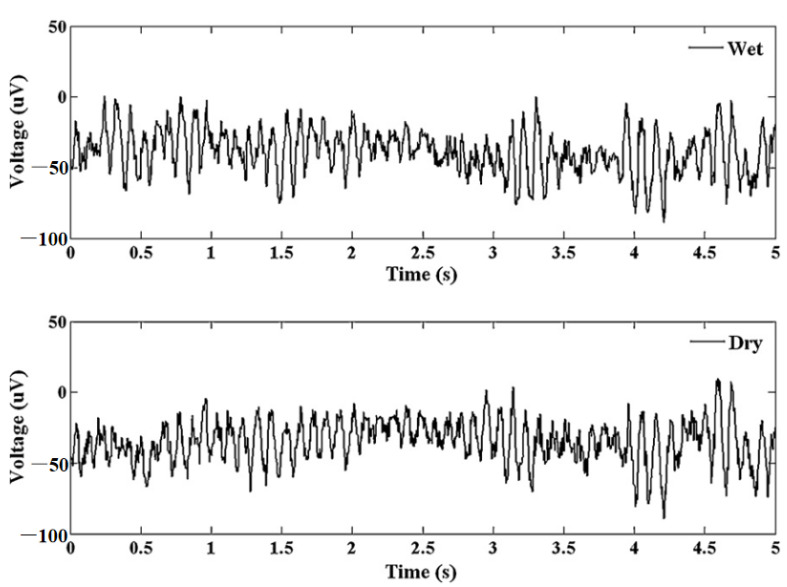
Alpha waves for wet electrode and MAE. Reprinted with permission from ref. [108].

**Figure 17:**
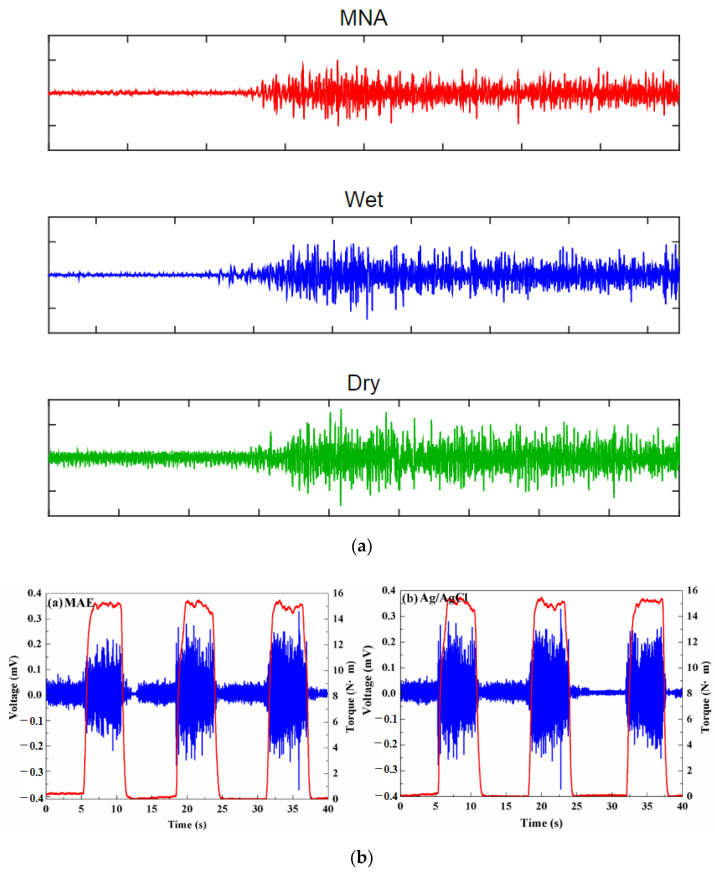

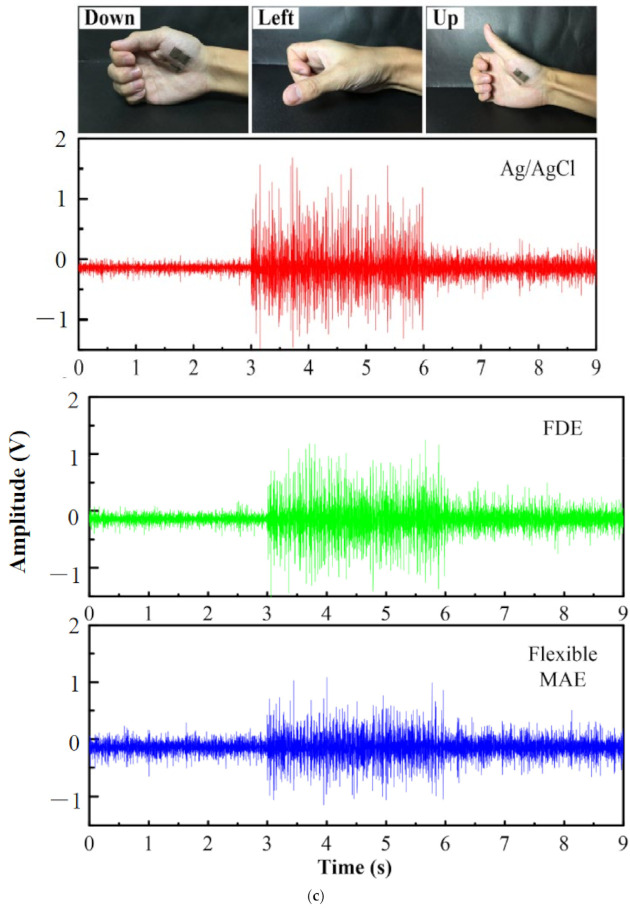

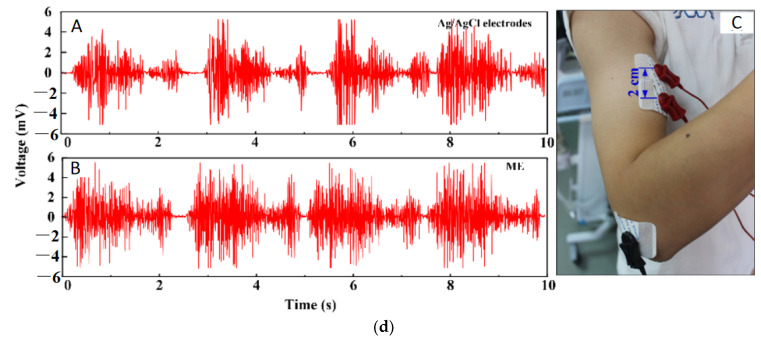
(**a**) EMG signal during the maximum voluntary contraction from the FCR before exercise [110]; (**b**) EMG signals recorded by MAE and Ag/AgCl electrodes [113]; (**c**) Muscle groups of thumbs recorded by MAE, flat dry electrode and wet electrode for comparison [111]; (**d**) EMG recorded by Ag/AgCl electrodes and MAE with recording positions on the arm [71].

ECG is vital for the continuous health monitoring of cardiovascular patients [15,104]. The effectiveness of MAE has been evaluated by various researchers [15,103,104,105,106,107] by comparing the measured static and dynamic ECG signal using a conventional wet electrode, and the results prove the potentiality of MAE as ECG measurement tools as shown in Figure 15. EEG represents brain activity and is used to diagnose epilepsy, brain death, insomnia, coma, etc. EEG signals are in the µV range and are relatively difficult to measure. Various researcherfs [34,106,108,109] have reported the effectiveness of MAE for EEG measurements as shown in Figure 16. EMG is a biomedical signal which characterizes muscular activity and is used to diagnose muscular dystrophy, neuromuscular disease, and as an investigative tool for kinesiology. The EMG signal recorded by MAE [71,110,111] showed that signals are more accurate than those using traditional wet electrodes. Some typical recorded EMG signals are shown in Figure 17.

### 5.3. Bio-Markers and Drug Monitoring

Sensing and monitoring bio-markers such as glucose, analytes, enzymes, hormones, proteins, etc., is crucial to holistically understanding the human physiological and psychological condition. Besides, drug monitoring is also important to regulate the dosage of highly sensitive drugs in humans. These monitoring systems need to be easy to use to ensure healthcare systems operate more smoothly and be more patient-friendly. Microneedle-based patient-friendly sensing and monitoring systems have attracted significant research interest, for example, a microneedle-based glucose monitoring system [114,115,116], a potassium ion monitoring system [117,118], an alcohol monitoring system [119], a body temperature monitoring system [120], chemical agents (toxic organophosphate (OP)) monitoring systems [121], and L-dopamine drug monitoring systems for Parkinson disease [122], etc.

### 5.4. Pediatrics

Delivering drugs to pediatric patients is a very challenging task [123]. It requires optimizing various factors for efficiently delivering the prescribed drugs. Traditionally, parenteral administration by hypodermic needle has been used, but some limitations exist such as pain, emotional trauma, needle stick injuries, risk of disease transmission, inefficiency in drug delivery, infections, and potential bio-hazards, etc. [90,123,124,125,126]. On the other hand, transdermal drug delivery using microneedles is promising to overcome these limitations [123]. Microneedles can bypass pain, emotional trauma, and needle stick injuries by delivering drugs by penetrating the stratum corneum without touching the nerve ending and blood capillaries [10,127]. The study by Mooney et al. reported that microneedle technology has been widely accepted by the pediatric population as it is associated with less pain [128]. The study of 66 children (age 9 to 15) by Salvador et al. also supported that children feel interested in microneedles technology and are enthusiastic to use microneedles in their future due to their safety and efficiency [129]. Recently, the microneedle array has received attention from numerous researchers as means of drug delivery to pediatric patients. Cormier et al. reported the treatment of enuresis in children by using a coated microneedles array [130]. J Gupta et al. and J. J. Norman et al. reported the effectiveness of microneedles for the treatment of type I diabetics in young children and teenagers [131,132]. The attitude of pediatricians and the general public toward microneedles as a means of drug delivery is also important for the widespread use of microneedles for pediatric patients. The study by Birchall et al. reported that most health care professionals and the general public consider that microneedle is safe for drug delivery to children [133].

### 5.5. Delivery of Peptides

Peptides are small strings of amino-acids and have many beneficial effects on human health [134]. Peptides can be used for the treatment of various diseases such as hypoglycemia [135], cancer treatment [136], and skin treatment [137], etc. Traditionally peptides are delivered through a hypodermic needle or oral administration. Oral administration has several limitations such as gastrointestinal irritation [138,139,140,141], teeth strain [142], and affecting liver function [140], etc. Transdermal delivery of peptides using microneedle can solve these problems and also solve the problem associated with a traditional hypodermic needle such as unregulated delivery, skin infections, pain, etc. [139,140]. Microneedle has been gaining a lot of attraction as an innovative way since it can enhance skin permeation and effectively transport a large variety of biomolecules across the skin by barely touching the skin [140,143], increasing drug adsorption by multifold times compared with a hypodermic needle [140]. For example, Mohammad et al. reported cosmeceutical peptides in the skin [137]. S. Li et al. reported an innovative way for peptide delivery by coating microneedle with drug-loaded nanoparticles which can be used for the co-delivery of multiple compounds with different properties [144]. K. van der Maaden et al. reported efficient induction of cytotoxic and T helper response using hollow microneedle [145]. Hairui Li et al. reported the administration of copper peptide by using a microneedle for skin regeneration and wound healing [139]. Amin Ghavami Nejad et al. reported the delivery of somatostatin receptors using a microneedle for preventing hypoglycemia of type I diabetic patient [135] etc.

### 5.6. Neural Implant

As the most complex part of the human body, studying the brain signal is the most challenging task for scientist and engineers [146]. With the advent of the well-established fabrication process, Si microneedles can be used as neural probes or tissue-penetrating electrodes for brain-signal monitoring [146,147,148,149]. Sang Heon Lee et al. and H. Sawahata et al. reported the neural implant of Si microneedle array for the in vivo electrophysiological recordings in small animals [150,151]. An implemented microneedle with a microfluidic channel was reported by Y. Son et al., M. Sakata et al., and M Shikida et al. to monitor the electrical signal generated from the brain activity [152,153,154]. A microneedle of 40 µm-width with CMOS array amplifier on the substrate was reported by Saxena et al. to increase the blood-brain barrier breach in probe implemented rats [155]. Dongxiao Yan et al. has reported metalized Si microneedle for peripheral brain interfacing. The study suggest that if the needle’s tip size is reduced to less than 10 um in diameter then it drastically lessens the tissue injury of the brain [156]. A. Fujishiro et al. [157], H Swata et al. [151], and Yuto Kita et al. [148] have reported a very small tip-sized (~7 µm, ~5 µm, ~3 µm respectively) Si microneedle for in vivo neural signal recording.

## 6. Future Prospects and Challenges

The microneedle has come along a long path toward bio-medical applications since its first introduction in 1970. It has created a new dimension in transdermal drug delivery with molecules of various sizes and opened a new scope and potential in other biomedical applications as well. Compared to conventional hypodermic needles, microneedles are less invasive, less painful, and more patient-friendly. Among various microneedles, in-plane Si microneedles have a well-established CMOS compatible microfabrication process which provides better control over needle geometry. In vitro studies [158,159,160] suggest that in the near future a comprehensive view of human health by heterogeneous monitoring systems on analytes, proteins, hormones, genes, etc., is achievable with a single microneedle array, which will be a great leap toward live patient monitoring [161]. High frequency, highly sensitive in vivo live drug monitoring using microneedles is also being investigated [122,162,163,164,165]. With such drug monitoring features, auto-regulation of drug delivery by determining the threshold drug level using close looped circuitry can be realized. Furthermore, point-of-care diagnostics can be achieved by the integration of microfluidic chips with microneedle arrays [165], which is an advance toward universal healthcare systems. The microneedle is also one of the best options as neural probes for long-time monitoring of bio-signals such as ECG, EEG, EMG, etc., and the day when microneedles are widely used to monitor real-life, for example, long-term home monitoring, brain-computer interface, neural disease diagnosis, etc., [15] is not far away. Microneedle technology has been widely accepted for drug delivery and diagnostics in pediatrics due to less pain [128], safety, and efficiency [129], which is desirable for use to treat diseases such as diabetics in young children and teenagers [131,132]. The perspective on microneedle-based drug delivery and diagnostics in pediatrics has been reported [166]. There are still some issues associated with the microneedle based drug delivery to the pediatric patients such as skin irritation, high cost, poor accuracy, lack of training of healthcare staff, and possible accidental use, etc. [123] The studies show that if these issues can be addressed properly, then there is a high prospect of the microneedle array as a means of drug delivery to the pediatric population.

Though the in-plane Si microneedle has shown its prospect in various biomedical applications, there are still challenges needing to be addressed. Human skin thickness is different due to the age, gender, and body fat of the patient [165], which could lead to partial or inappropriate insertion into the skin [165]. This problem needs to be addressed by the suitable geometric shape of the microneedle supported by numerical analysis and appropriate design. Sterilizing the microneedle for repeated uses [38] is another issue to be addressed. There is a high chance of skin infection, inflammation, irradiation, swelling, and erythema caused by repeated penetration [38,165,167]. Evaluation of the clinical impact of the repeated application of the microneedle array is required, and easy sterilization and intact removal from the skin are desirable. Short-term effects and long-term stability associated with microneedle insertion are another major challenge [167] and demand an extensive investigation to develop solutions, for example, suitable biocompatible coating materials or transient coating [168] to reduce these effects of insertion and enhance long-term stability. One more challenge exists in expanding the in-plane Si microneedle in sensor applications from bio-signal/biomarker detection to continuous monitoring [165]. Functionalization of the Si microneedle with aptamer-based polymer or other nanomaterial has proven to be a promising solution [169,170,171,172] and needs rigorous study for optimization. Precise control in drug delivery or pinpointed localization during sample collection [38,165,167] is another crucial challenge, which requires extra care in the design and fabrication of the microneedle. In summary, it is evident that in-plane Si microneedles can play a pivotal role in the biomedical sector if these challenges can be addressed.

## 7. Conclusions

The use of microneedles as a means of biomedical tools has a great prospect due to its advantages over traditional hypodermic needles including minimum invasiveness, less pain, low material and fabrication cost, precise geometry control, and so on. In-plane and out-of-plane microneedles made of different materials such as metal, polymer, and Si have been investigated. Among these microneedles, in-plane Si microneedles excel by their inherent characteristics of biocompatibility, CMOS compatible fabrication, a wide range of needle length, and capability of integration with other sensing and microfluidic components etc. In this paper, major fabrication techniques used to make in-plane Si microneedles, theoretical and computation analyses, as well as experimental characterizations of needle strength and microfluidic flow for drug delivery, were reviewed. Furthermore, applications, challenges, and future prospects of in-plane Si microneedles have also been discussed. Research and investigation have indicated that these devices are promising for a wide range of biomedical applications including drug delivery, sample collections, medical diagnostics, bio-sensing, and bio-signal monitoring.

## Figures and Tables

**Table 1:** Some representative in-plane microneedle designs.

Sl.	Reference	Schematic
1.	Reprinted with permission from ref. [16]	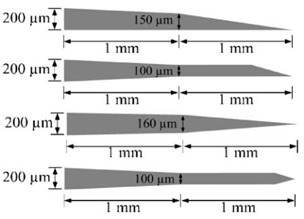
2.	Reprinted with permission from ref. [3]	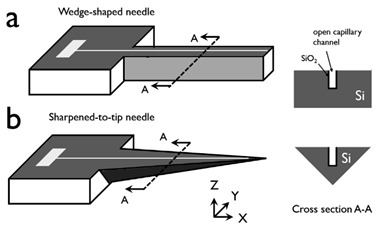
3.	Reprinted with permission from ref. [39]	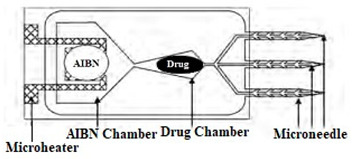

**Table 2:** Area moment of inertia *I* for microneedles with different shapes [2,19].

Types of Microneedle	Moment of Inertia	Definition of the Term
Circular	$I=\frac{1}{64}\pi \left(D^{4}-d^{4}\right)$	*D* and *d* are the outer and inner diameters of the needle, respectively.
Rectangular	$I=\frac{1}{12}\left(BH^{3}-bh^{3}\right)$	*B* and *H* are outer width and thickness, and *b* and *h* are the inner width and thickness.
Square	$I=\frac{1}{12}\left(H^{4}-h^{4}\right)$	*H* and *h* are the outer and inner dimensions, respectively.
Solid Conical	$I=\frac{Dy^{3}}{396}$	*y* is the length of the conical section, and *D* is the diameter.

**Table 3:** Experimental vs. computed flow rate. The computed flow rate is determined by estimating the entrance length using equation (15) and followed by applying equation (20) to correlate pressure loss with flow rate.

SL. No	Needle Style	Number of Tests	Avg. Measured Flow Rate (cc/sec)	Computed Flow Rate (cc/sec)	Error (%)	Reynold Numbers
1.	Bent, 90°	4	0.082 ± 0.004	0.088	7.3	738
2.	Reinforced	9	0.040 ± 0.004	0.040	0.0	503
3.	Fillet	2	0.070 ± 0.01	0.083	17.9	688
4.	Double Channel	1	0.032	0.o34	6.2	260

## Data Availability

Not applicable.
